# Effects of Progressive Muscle Relaxation and Breathing Exercises with Music on Stress Levels and Bio-Psycho-Social Responses of Nursing Students in Clinical Practice: A Randomized Controlled Trial

**DOI:** 10.1192/j.eurpsy.2025.2003

**Published:** 2025-08-26

**Authors:** N. Karabulut, Ç. Yüksel

**Affiliations:** 1 Nursing, Ankara University; 2 Nursing, University of Health Sciences, Ankara, Türkiye

## Abstract

**Introduction:**

Clinical practice involves many stress factors for nursing students. Stress in clinical practice causes positive or negative outcomes for students. Students show physical, emotional and behavioural reactions to stress. Therefore, progressive muscle relaxation and breathing exercises with music can be a method that students can easily learn and apply, reducing stress and negative bio-psycho-social responses.

**Objectives:**

This study aimed to examine the effects of progressive muscle relaxation and breathing exercises accompanied by music on the stress levels of undergraduate nursing students and their bio-psycho-social responses to stress.

**Methods:**

This randomized controlled study was conducted at a university in Turkey with ethics committee approval. A total of 154 undergraduate nursing students were randomized, 77 in the intervention group and 77 in the control group. 44 of the students were sophomores, 52 were third-year students, and 58 were fourth-year students. Progressive muscle relaxation and breathing exercises were applied to the intervention group for six weeks with music. No intervention was applied to the control group during the research period. Research data were collected using the Personal Information Form, the Perceived Stress Scale for Nursing Students, and the Biopsychosocial Response Scale for Nursing Students. The scales were applied to the intervention and control groups before the exercises, at the end of the six-week exercises, and two weeks after the exercises ended. Data were collected between October 2022 and January 2023. Shapiro-Wilk test was used in the analysis of normality of data; Independent Samples t-test, Dependent Samples t-test, and Analysis of Variance were used in the analysis of variables.

**Results:**

In the pre-test measurements of the groups, it was found that there was no significant difference in terms of the students’ stress levels in clinical practice and bio-psycho-social response scores (p>0.05). In the post-test and follow-up measurements, it was seen that the stress and bio-psycho-social response scores were significantly lower in the intervention group than in the control group (p<0.05). There was also a significant group*time interaction between the groups in terms of stress and bio-psycho-social response scores (p <0.05).

**Conclusions:**

The findings showed that the intervention helped students reduce their stress levels and negative bio-psycho-social responses to stress, and the effects were found to be sustained in the short term. These positive results are promising in the use of progressive muscle relaxation and breathing exercises with music as an effective and easy method to reduce the stress level and negative physical, emotional and behavioral responses to stress in the clinical practice of undergraduate nursing students.

**Disclosure of Interest:**

None Declared

